# Expression of Autophagy Markers LC3B, LAMP2A, and GRP78 in the Human Kidney during Embryonic, Early Fetal, and Postnatal Development and Their Significance in Diabetic Kidney Disease

**DOI:** 10.3390/ijms25179152

**Published:** 2024-08-23

**Authors:** Ivan Brdar, Anita Racetin, Ivo Jeličić, Katarina Vukojević, Ljiljana Vučković, Dragan Ljutić, Mirna Saraga-Babić, Natalija Filipović

**Affiliations:** 1 Emergency Department, University Hospital of Split, Spinčićeva 1, 21000 Split, Croatia; ivan_brdar@yahoo.com; 2 Department of Anatomy, Histology and Embryology, University of Split School of Medicine, Šoltanska 2, 21000 Split, Croatia; anita.racetin@mefst.hr (A.R.); kvukojev@gmail.com (K.V.); msb@mefst.hr (M.S.-B.); 3 Internal Medicine Department, Nephrology and Haemodialysis Division, University Hospital of Split, Šoltanska 1, 21000 Split, Croatia; ivo.jelicic@gmail.com (I.J.); dragan.ljutic@yahoo.com (D.L.); 4 Department of Anatomy, School of Medicine, University of Mostar, Bijeli Brijeg bb, 88000 Mostar, Bosnia and Herzegovina; 5 Clinic for Pathology and Citology, Clinical Center of Montenegro, 81101 Podgorica, Montenegro; ljvuckovic@gmail.com; 6 Department of Histology and Embryology, Medical Faculty, University of Montenegro, 81101 Podgorica, Montenegro

**Keywords:** human embryo, early human development, diabetic kidney disease, chronic kidney disease, autophagy, LC3B, LAMP2A, GRP78

## Abstract

Autophagy is the primary intracellular degradation system, and it plays an important role in many biological and pathological processes. Studies of autophagy involvement in developmental processes are important for understanding various processes. Among them are fibrosis, degenerative diseases, cancer development, and metastasis formation. Diabetic kidney disease is one of the main causes of chronic kidney disease and end-stage renal failure. The aim of this study was to investigate the immunohistochemical expression patterns of LC3B, LAMP2A, and GRP78 during different developmental stages of early-developing human kidneys and in samples from patients with type II diabetes mellitus. During the 7/8th DW, moderate expression of LC3B and LAMP2A and strong expression of GRP78 were found in the mesonephric glomeruli and tubules. In the 9/10th DW, the expression of LC3B and LAMP2A was even more pronounced in the mesonephric tubules. LC3B, LAMP2A, and GRP78 immunoreactivity was also found in the paramesonephric and mesonephric ducts and was stronger in the 9/10th DW compared with the 7/8th DW. In addition, the expression of LC3B, LAMP2A, and GRP78 also appeared in the mesenchyme surrounding the paramesonephric duct in the 9/10th DW. In the 15/16th DW, the expression of LC3B in the glomeruli was weak, that of LAMP2A was moderate, and that of GRP78 was strong. In the tubuli, the expression of LC3B was moderate, while the expression of LAMP2A and GRP78 was strong. The strongest expression of LC3B, LAMP2A, and GRP78 was observed in the renal medullary structures, including developing blood vessels. In postnatal human kidneys, the most extensive LC3B, LAMP2A, and GRP78 expression in the cortex was found in the epithelium of the proximal convoluted tubules, with weak to moderate expression in the glomeruli. The medullary expression of LC3B was weak, but the expression of LAMP2A and GRP78 was the strongest in the medullary tubular structures. Significantly lower expression of LC3B was found in the glomeruli of the diabetic patients in comparison with the nondiabetic patients, but there was no difference in the expression of LC3B in the tubule–interstitial compartment. The expression of LAMP2A was significantly higher in the tubule–interstitial compartments of the diabetic patients in comparison with the nondiabetic patients, while its expression did not differ in the glomeruli. Extensive expression of GRP78 was found in the glomeruli and the tubule–interstitial compartments, but there was no difference in the expression between the two groups of patients. These data give us new information about the expression of LC3B, LAMP2A, and GRP78 during embryonic, fetal, and early postnatal development. The spatiotemporal expression of LC3B, LAMP2A, and GRP78 indicates the important role of autophagy during the early stages of renal development. In addition, our data suggest a disturbance in autophagy processes in the glomeruli and tubuli of diabetic kidneys as an important factor in the pathogenesis of diabetic kidney disease.

## 1. Introduction

Autophagy is the primary intracellular degradation system, which is the essential molecular process by which the recycling of cytoplasmic elements in lysosomes creates the preconditions for preserving cell homeostasis [1]. It is considered an evolutionarily preserved mechanism necessary for the survival and restoration of cells in critical conditions [2,3]. There are three forms of autophagy: macroautophagy, microautophagy, and chaperone-mediated autophagy (CMA). Macroautophagy is considered the main form of autophagy and is the best researched [2]. It involves the formation of a double-membrane vesicle (autophagosome), which separates a portion of the cytoplasm with organelles or soluble substances. The autophagosome then merges with a lysosome to form an autolysosome in which the contained substances are degraded [2,4]. In microautophagy, lysosomal membrane invaginations and protrusions fully encompass one part of the cytoplasm with small cytoplasmic substances [5]. CMA does not include membrane structures and is highly specific. Along with cytosolic proteins, chaperon proteins are translocated over the lysosome membrane and into the lumen, where protein unfolding and degradation take place [3]. The surface of the autophagy vesicles’ membrane contains LC3B, which is crucial for developing autophagosomes and is being used as an autophagy marker [6]. Converted from its cytosolic form (LC3-I) to its membrane-lipidated form (LC3-II), LC3, a soluble protein, is a structural component of both the inner and outer layers of the autophagosome membrane [7,8]. In their study, Xin et al. (2016) showed that the activation of autophagy has a protective role in human podocytes against high glucose-induced insulin resistance and cell injury [9]. LC3BII expression was markedly reduced in high-glucose-cultured human podocytes compared with control groups, suggesting suppressed autophagy flux. An increase in LC3BII levels has been reported with the use of rapamycin, a potent inducer of autophagy. The autophagy level change was confirmed by both western blot and immunofluorescent staining. Also, with the activation of autophagy, there was an increase in glucose intake and phosphorylation of insulin receptors, indicating autophagy’s effect on insulin sensitivity [9].

The transmembrane protein LAMP-2A plays the role of a receptor on lysosomes [1,3]. Glycoproteins known as lysosome-associated membrane proteins 1 and 2 (LAMP-1, LAMP-2) account for almost half of the membrane proteins found on lysosomes [10]. Although there are various known LAMP-2 isoforms (LAMP-2A, LAMP-2B, and LAMP-2C), the available data indicate that only the LAMP-2A variation is significant for CMA [11]. Napolitano et al. (2015) conducted a study with the aim of better understanding the distribution of this receptor in renal anomalies [12]. Expression and localization of LAMP2A were inappropriate in cystine transporter cystinosis (CTNS) deficient cells, and degradation of CMA substrate was defective in Ctns −/− mice [12]. These mice have an apical distribution of LAMP2A in the cells of the proximal tubules, which is absent in the basal parts of the cells [13]. These findings suggest that renal injury in cystinosis may be caused by defective LAMP2A trafficking and consequent inhibition of CMA activity [14].

Glucose-regulated protein 78 (GRP78), a representative of the heat shock protein 70 (HSP70) superfamily [15], is an endoplasmic chaperon protein that plays a role in unfolded protein response (UPR) during endoplasmic reticulum (ER) stress, intending to return to ER homeostasis [16]. In response to ER stress, autophagy is activated in mammalian cells as a defense mechanism for cell survival. GRP78 plays a role in the formation of autophagosomes and is necessary for stress-induced autophagy [17]. Its expression is elevated in DKD but also in other renal disorders such as renal fibrosis and idiopathic nephrotic syndrome [13,18]. Recent studies have pointed to the possibility of translocation of GRP78 to the cell surface, where it plays a role in the process of diseases such as cancer and immune and inflammatory conditions. It has been demonstrated that high glucose levels promote cell surface expression of GRP78 [19]. It is an ER stress-dependent process via a DnaJ-like protein 1 that acts as a co-chaperone for the translocation of GRP78 to the cell surface, where it has profibrotic activity [19].

Autophagy appears to play an important role in many biological and pathological processes. Studies have shown that autophagy has a role in different developmental processes. It is important for mesenchymal-to-epithelial transition (MET) and epithelial-to-mesenchymal transition (EMT), cell fate decisions, and stem cell maintenance [20]. In addition, it has a role in induced pluripotent cell reprogramming, but also in the maintenance of stemness in cancer cells [20]. The knowledge about the role of autophagy in developmental processes is important. It helps to explain different pathological processes, such as fibrosis, degenerative diseases, cancer development, and metastasis formation [20].

The development of the mammalian kidney during embryonic life involves reciprocal induction between the ureteric bud and the metanephric mesenchyme, which are two intermediate mesenchymal progenitors that reciprocally induce each other during the multistage process [21]. Key contributions in nephrogenesis are MET [22] and a reverse process of the EMT [23]. Three successive, partly overlapping generations of structures are formed during human embryonic kidney development [24,25]. Pronephros is rudimentary, has no function, and disappears at the end of week four. Mesonephros begins development late in week four and decays at the end of the second month. Metanephros, or definitive kidney, appears at week five. Its excretory units develop from the methanephric mesoderm. Collecting ducts of the definitive kidney develop from the ureteric bud and penetrate the methanephric tissue [24]. Molecular signaling from the ureteric bud induces MET in the methanephric mesenchyme, causing the formation of the renal vesicles, which then quickly lengthen, forming a comma-shaped body, which elongates into an S-shaped loop, and at the medial end, a tangle of blood capillaries grows into them. As the loop is elongating, a nephron is formed. A complex interaction of various signaling systems is involved in the regulation of these processes. Increasing evidence points to the role of autophagy in renal development [26]. Using a single-cell RNA sequencing approach, Wen-Jin et al. (2021) revealed the dynamics of the expression of different ATG-proteins in various populations of a human fetal kidney and determined the relevance of autophagy in the development and cellular heterogeneity of early human fetal kidney [27].

Autophagy works in both cell protection and cell death. There is a very complex relationship between autophagy and apoptosis. Autophagic programmed cell death was initially described in tissues undergoing active development. In mammals, autophagy is involved in specific cytosolic rearrangements necessary for proliferation and differentiation during embryogenesis and postnatal development [28]. Zhang et al. (2017) showed that a decrease in the level of nephrine, a marker of podocyte differentiation, indicated a weakening of podocyte differentiation when autophagy activity was reduced [29]. Gamma-secretase inhibitor (DAPT) inhibited the Notch signaling pathway in embryonic kidneys, and autophagy decreased significantly with impaired differentiation. Namely, podocytes in the presence of DAPT had fewer autophagosomes than normal podocytes and reduced levels of LC3BII. Adding rapamycin to increase the autophagy activity in DAPT-treated cells resulted in the recovery of nephrine. These findings suggest the involvement of autophagy in the developmental processes of the kidneys.

However, further studies on the expression of other autophagy and ER stress markers in the embryonal development of the human kidney are needed to completely understand their role.

The prevalence of diabetes in the world has been rising markedly in recent decades, especially in developed countries [30]. Diabetic kidney disease (DKD) is the most common and severe complication of diabetes, strongly associated with an increasing number of patients with chronic kidney disease (CKD) whose prevalence is estimated at 10% of the world’s population and is the leading cause of end-stage renal failure (ESRD) [25,31]. It occurs due to microvascular complications that cause kidney damage and is characterized by persistent proteinuria and declining renal function. DKD is represented by specific histopathological changes: the basal membrane’s progressive thickening, mesangial matrix expansion, and glomerulosclerosis [32]. DKD’s emergence and progression process is very complex and involves several different pathways and factors, and it has not been fully understood yet. The activation of autophagy plays a vital role in the survival of kidney cells in critical conditions, such as hypoxia and oxidative stress [33]. Inadequate autophagy can ultimately lead to cellular damage and plays a role in the pathogenesis of diabetic nephropathy [33]. Previous research has shown that autophagy was suppressed in the proximal tubules of rats with streptozocin (STZ)-induced early diabetes [34]. Similar results were also shown by other research on early diabetic rats; autophagy was inhibited in distal tubules with a reversion of inhibition after insulin administration [35]. In addition, Yamahara and colleagues showed the mTORC1 activation and suppression of autophagy (with the accumulation of p62 protein) in the proximal tubule epithelial cells of renal biopsy specimens from patients with diabetes mellitus type 2 (DM2) [36]. Excess energy in DN is associated with the disappearance of the protective action of autophagy in the kidney. Consequently, damaged proteins and organelles accumulate, and DN progresses. Activating autophagy has a whole range of positive effects on the kidney. It positively affects inflammatory processes and fibrosis and slows down the further development of DKD [37]. It may be an effective therapy for DKD.

Additional studies are necessary to elucidate the role of autophagy in DKD pathophysiology in order to find new potential therapeutic targets. Since the activation of developmental expression patterns of different regulatory molecules is a characteristic of many pathological conditions [38,39], including CKD, these processes may lead to the progression of damage but may also play a role in regeneration and repair [40].

Therefore, to understand the role of autophagy and ER stress in kidney organogenesis and the pathogenesis of CKD, in this study, we aimed to investigate the expression patterns of LC3B, LAMP2A, and GRP78 during different developmental stages of early developing human kidneys and in samples from patients with diabetes mellitus, as one of the major causes of CKD.

## 2. Results

We studied the expression of autophagy and endoplasmic reticulum stress markers during the early development of kidneys in humans. We found expression of all the studied factors—LC3B, LAMP2A, and GRP78—in different renal structures during the embryonal and fetal periods.

### 2.1. LC3B Immunoexpression during Early Renal Development

**Figure 1:**
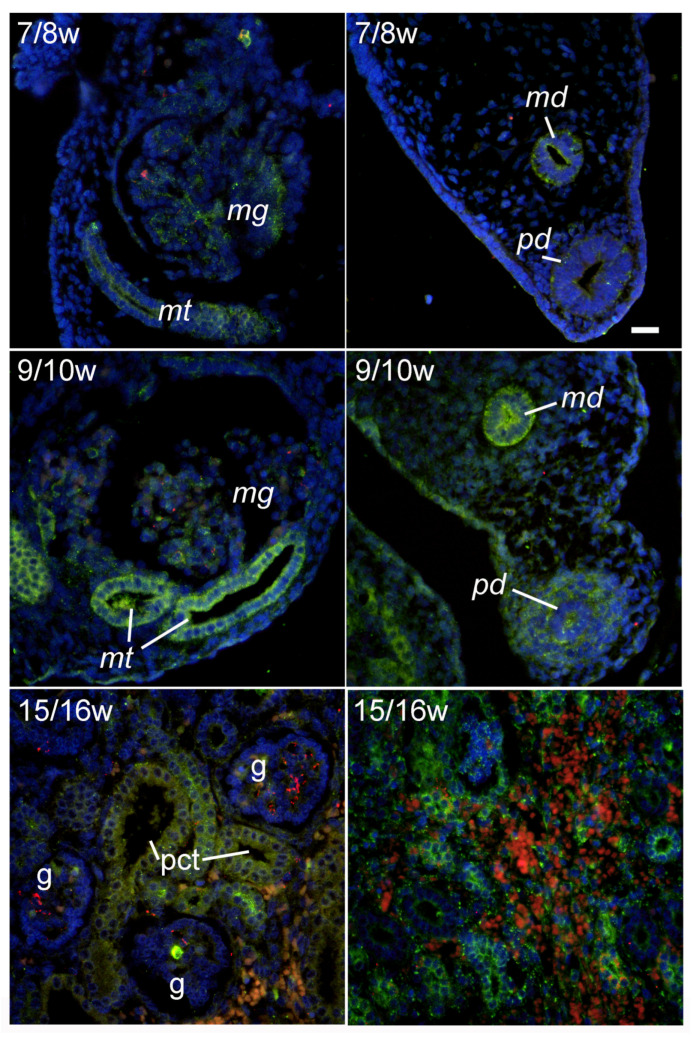
Immunohistochemical expression of LC3B (green) in developing human kidneys and surrounding structures: 7/8w—developmental weeks 7/8; mg—mesonephric glomerulus; mt—mesonephric tubuli; md—mesonephric duct; pd—paramesonephric duct; 9/10w—developmental weeks 9/10; 15/16w—developmental weeks 15/16 (left panel—cortex, right panel—medulla); g—glomerulus; pct—proximal convoluted tubuli. Objective magnification ×40.

During the 7/8th DW, moderate expression of LC3B was found in mesonephric glomeruli and tubules. Similarly, in the 9/10th DW, expression of LC3B was also found in the mesonephric glomeruli, while LC3B immunoreactivity was even more pronounced in mesonephric tubules. LC3B immunoreactivity was also found in the paramesonephric and mesonephric ducts and was stronger in the 9/10th DW compared with the 7/8th DW. In addition, moderate LC3B expression was also found in the mesenchyme surrounding the paramesonephric duct in the 9/10th DW. In the fetal kidney, in the 15/16th DW, the expression of LC3B was present in the renal cortex, where it was weak in the glomeruli and moderate in the developing tubules. Substantially stronger LC3B expression during the 15/16th DW was observed in the renal medullar structures, including the developing blood vessels and tubular structures (Figure 1).

### 2.2. LAMP2A Immunoexpression during Early Renal Development

**Figure 2:**
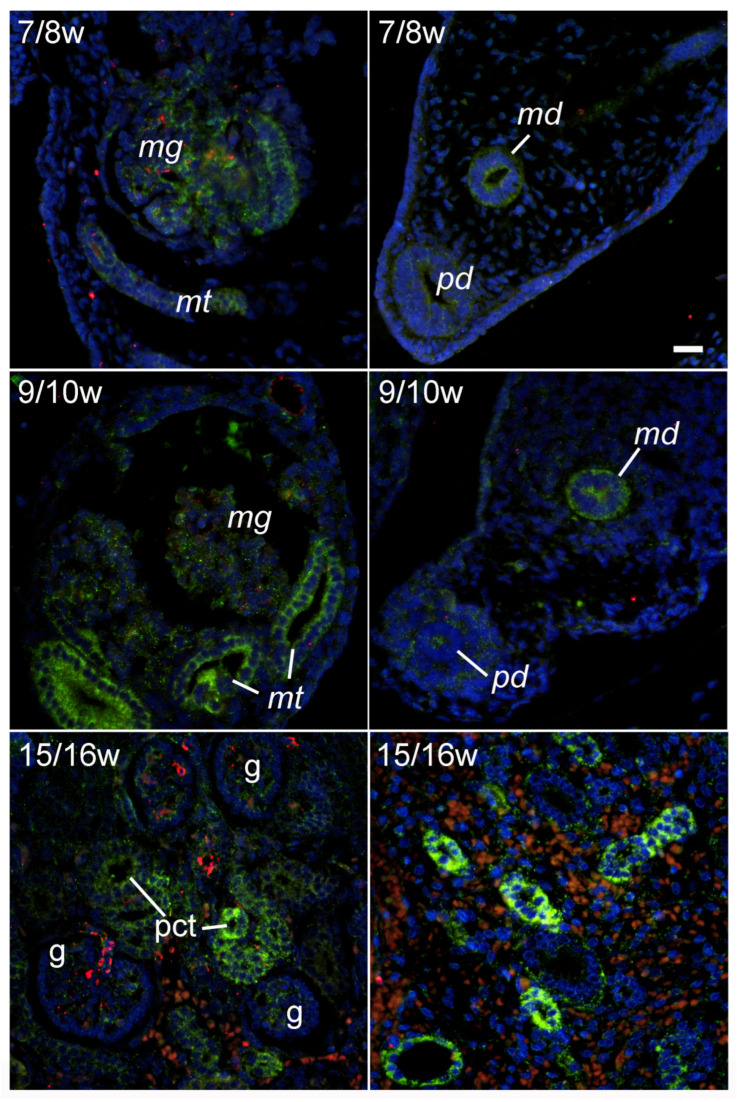
Immunohistochemical expression of LAMP2A (green) in developing human kidneys and surrounding structures: 7/8w—developmental weeks 7/8; mg—mesonephric glomerulus; mt—mesonephric tubuli; md—mesonephric duct; pd—paramesonephric duct; 9/10w—developmental weeks 9/10; 15/16w—developmental weeks 15/16 (left panel—cortex, right panel—medulla); g—glomerulus; pct—proximal convoluted tubuli. Objective magnification ×40.

The pattern of LAMP2A immunoexpression during renal development was similar to that of LC3B. During the 7/8th DW, moderate LAMP2A expression was found in the mesonephric glomeruli and tubuli, and in the 9/10th DW, the expression of LAMP2A was also found in the mesonephric glomeruli and tubuli, but it was even more pronounced in comparison to the 7/8th DW. In addition, LAMP2A immunoreactivity was also found in the paramesonephric and mesonephric ducts, being stronger in the 9/10th DW compared with the 7/8th DW. Similar to the LC3B, LAMP2A expression was also found in the mesenchyme surrounding the paramesonephric duct during the 9/10th DW. In the fetal kidney, in the 15/16th DW, moderate expression of LAMP2A was observed in the glomeruli, while strong LAPM2A immunoreactivity was found in the developing tubuli. The LAMP2A expression during the 15/16th DW was very strong in the medullary tubular structures, and it was moderate in the developing medullary blood vessels (Figure 2).

### 2.3. GRP78 Immunoexpression during Early Renal Development

**Figure 3:**
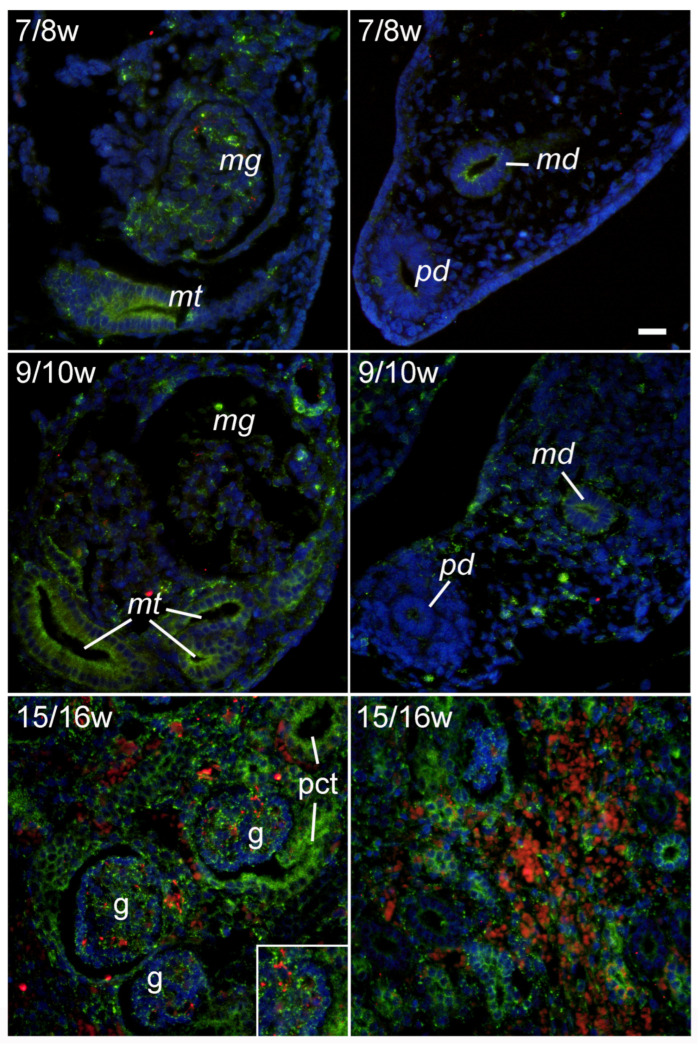
Immunohistochemical expression of GRP78 (green) in developing human kidneys and surrounding structures: 7/8w—developmental weeks 7/8; mg—mesonephric glomerulus; mt—mesonephric tubuli; md—mesonephric duct; pd—paramesonephric duct; 9/10w—developmental weeks 9/10; 15/16w—developmental weeks 15/16 (left panel—cortex, right panel –medulla); g—glomerulus; pct—proximal convoluted tubuli. Objective magnification ×40.

During the 7/8th DW, the expression of the ER stress marker GRP78 was strong in the mesonephric glomeruli and tubule, which similarly continued in the 9/10th DW. GRP78 immunoreactivity was also observed in paramesonephric and mesonephric ducts, but, in opposite to LC3B and LAMP2A, GRP78 expression was more pronounced in mesonephric, in comparison to paramesonephric duct. During the 9/10th DW, GRP78 immunoreactivity also appeared in the mesenchyme surrounding the mesonephric duct. In the fetal kidney, during the 15/16th DW, strong GRP78 expression was found in all cortical and medullary structures, including glomeruli, tubular structures, and developing blood vessels (Figure 3).

### 2.4. Expression of LC3B, LAPM2A, and GRP78 in Postnatal Human Kidney

**Figure 4:**
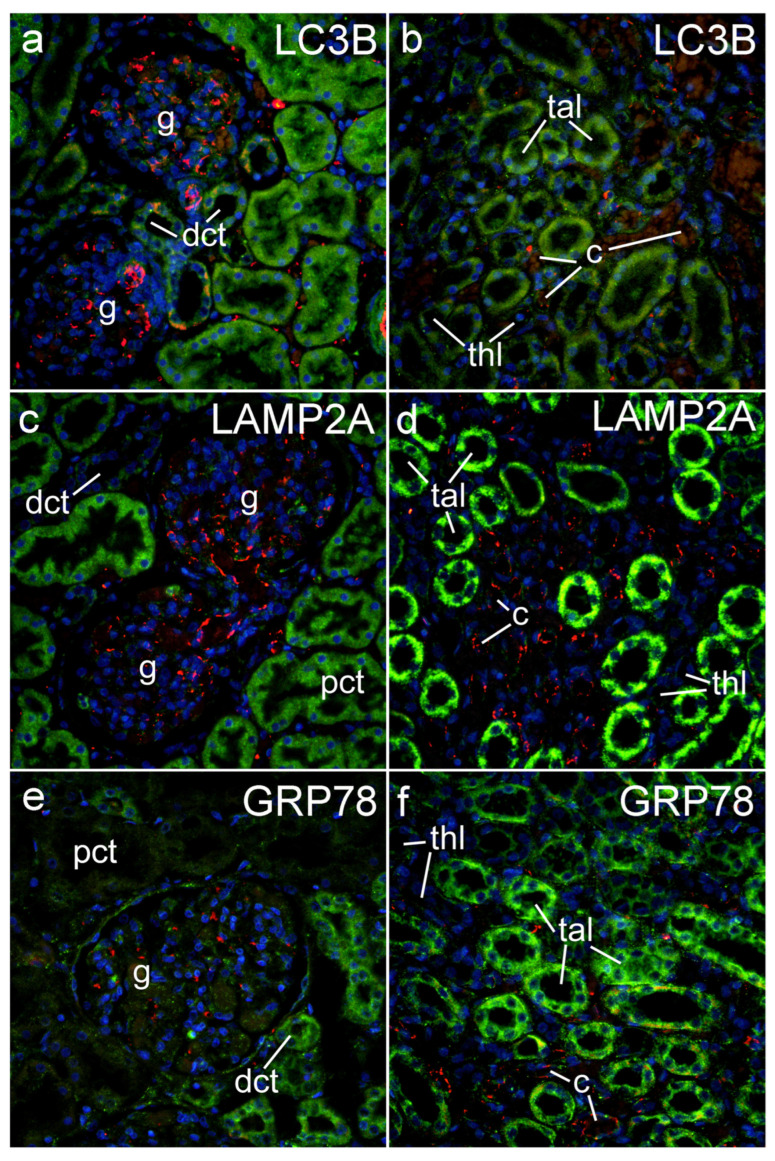
Immunohistochemistry expression of LC3B, LAPM2A, and GRP78 in postnatal human kidney: Green—protein of interest ((**a**,**b**)—LC3B; (**c**,**d**)—LAPM2A; and (**e**,**f**)—GRP78); red—CD31, endothelial marker; blue—DAPI. Left panel (**a**,**c**,**e**)—cortex; right panel (**b**,**d**,**f**)—medulla; g—glomerulus; pct—proximal convoluted tubule; dct—distal convoluted tubule; c—capillary; thl—thin limb of the loop of Henley; tal—thick ascending limb of the loop of Henley. Objective magnification ×40.

In addition, we used immunohistochemistry to study the expression of LC3B, LAPM2A, and GRP78 in postnatal human kidney (Figure 4). The most extensive LC3B expression in the cortex was found in the epithelium of the proximal convoluted tubules. However, weak LC3B expression was found in the glomerular cells (but not present in Bowman’s capsule cells), distal convoluted tubules, and collecting ducts. In the medulla, LC3B expression was moderate in the collecting duct cells and thin limb and blood capillary wall cells. LAMP2A immunoreactivity in the renal cortex was the strongest in the proximal convoluted tubules, and weak in the glomerular cells, distal tubules, and the collecting duct epithelia. It was not present in Bowman’s capsule cells. In the medulla, LAMP2A expression was found to be very strong in the collecting ducts and weak to nonexisting in the other medullary structures. In the cortex of the postnatal human kidney, the immunoexpression of GRP was strong in the collecting duct cells and moderate in the glomerular cells. In contrast to the other two studied molecules, GRP78 was moderately expressed in Bowman’s capsule cells. In the medulla, GRP78 expression was the strongest in the collecting duct cells, but it was also moderately expressed in the other cells present, including the thin and thick limbs of the loop of Henley and blood capillary wall cells. 

### 2.5. Expression of LC3B, LAMP2A, and GRP78 in Renal Tissues of Diabetic Patients

The patients’ age, sex, and serum creatinine data are presented in Table 1.

#### 2.5.1. Expression of LC3B in Renal Tissues of Diabetic Patients

**Figure 5:**
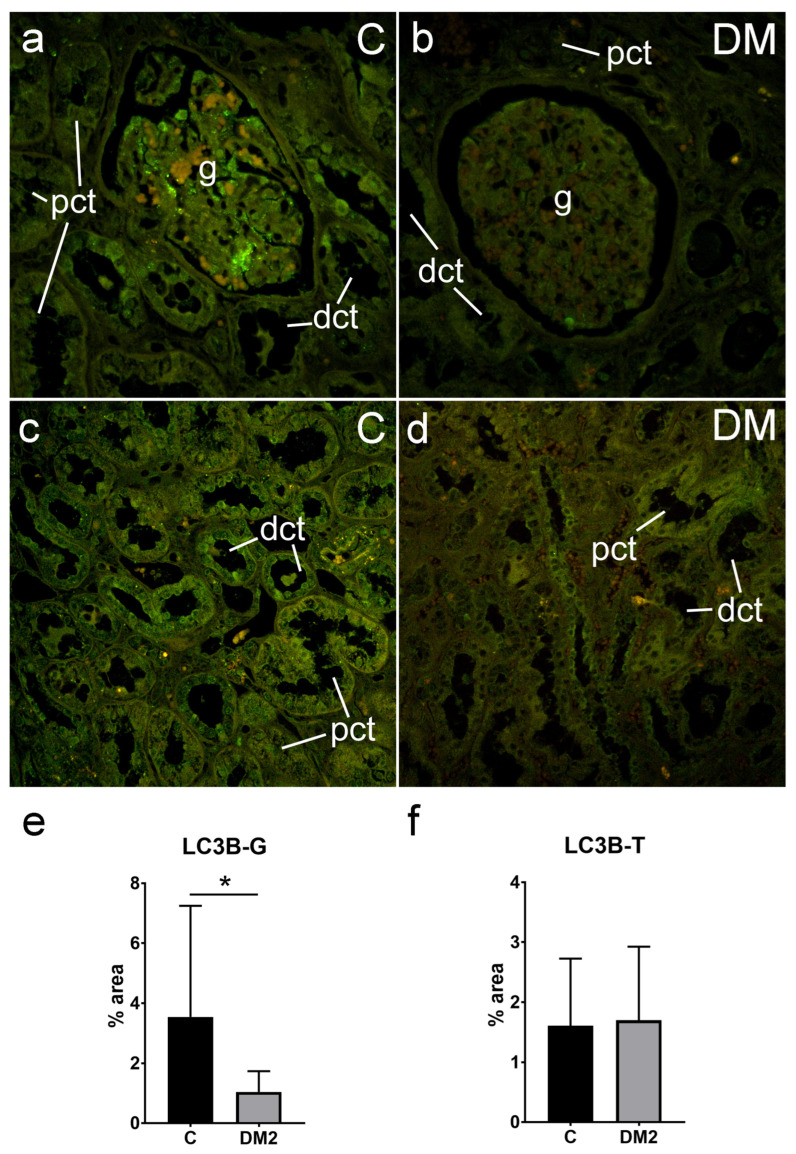
Immunohistochemistry expression of LC3B (green) in renal tissues of nondiabetic and diabetic patients: C—control; DM2—diabetic patients; (**a**,**b**)—glomeruli (objective magnification ×40); (**c**,**d**)—tubule–interstitial compartment (objective magnification ×20); (**e**,**f**)—results of the LC3B analysis—immunofluorescence signal was expressed as tissue section percentage area (% area)—in glomeruli (LC3B-G) and tubuli (LC3B-T) and compared using unpaired *t*-test. * Statistically significant difference between the indicated groups at *p* < 0.05. Grey column—diabetic patients (DM2); black column—control group of patients; mean (column) ± standard deviation (whisker) is presented. g—glomerulus; pct—proximal convoluted tubule; dct—distal convoluted tubule.

We explored the expression of LC3B in the kidney tissue of diabetic and nondiabetic patients. LC3B immunoreactivity was moderate to weak. We only found strong LC3B expression occasionally in the glomeruli of nondiabetic patients. Image analysis revealed a significantly lower expression of LC3B in the glomeruli of the diabetic patients (1.04 ± 0.69%) in comparison to the nondiabetic patients (3.54 ± 3.71%) (*p* = 0.003 for logarithmically transformed data; Figure 5e). However, there was no difference in the expression of LC3B in the tubule–interstitial compartment of the cortex between the two groups (diabetic—1.70 ± 1.23%; control—1.61. ± 1.12: *p* = 0.656 for logarithmically transformed data; Figure 5f). 

#### 2.5.2. Expression of LAMP2A in Renal Tissues of Diabetic Patients

**Figure 6:**
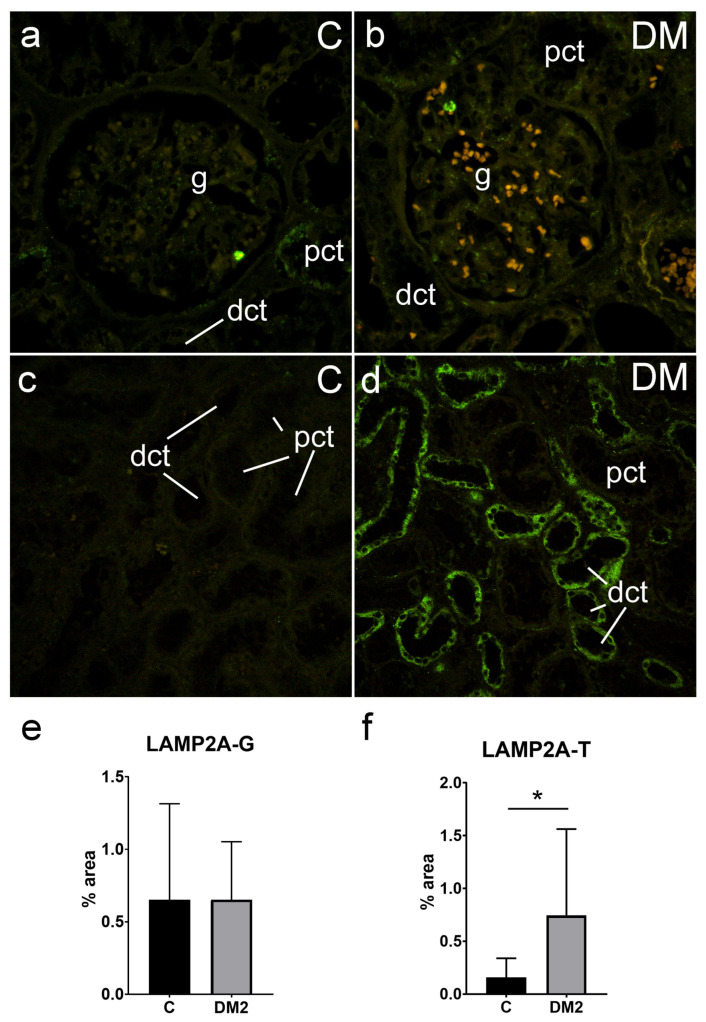
Immunohistochemistry expression of LAMP2A (green) in renal tissues of nondiabetic and diabetic patients: C—control; DM2—diabetic patients; (**a**,**b**)—glomeruli (objective magnification ×40); (**c**,**d**)—tubule–interstitial compartment (objective magnification ×20); (**e**,**f**)—results of the LAMP2A analysis—immunofluorescence signal was expressed as tissue section percentage area (% area)—in glomeruli (LAMP2A-G) and tubuli (LAMP2A-T) and compared using unpaired *t*-test. * Statistically significant difference between the indicated groups at *p* < 0.05. Grey column—diabetic patients (DM2); black column—control group of patients; mean (column) ± standard deviation (whisker) is presented. g—glomerulus; pct—proximal convoluted tubule; dct—distal convoluted tubule.

In addition, we studied the expression of LAMP2A in the kidney tissues of diabetic and nondiabetic patients. LAMP2A immunoreactivity was mostly weak in the glomeruli, as well as in the tubule–interstitial compartment. However, we found strong LAMP2A immunoreactivity in the tubuli of diabetic patients. The expression of LAMP2A was significantly higher in the tubule–interstitial compartment of the diabetic patients (0.75 ± 0.82%) in comparison to the nondiabetic patients (0.16 ± 0.18% *p* < 0.05; Figure 6f). There was no difference in the expression of LAMP2A in the glomeruli between the two groups of patients (diabetic 0.65 ± 0.40%; vs. control group 0.65 ± 0.66; *p* = 0.012 for logarithmically transformed data; Figure 6e).

#### 2.5.3. Expression of GRP78 in Renal Tissues of Diabetic Patients

**Figure 7:**
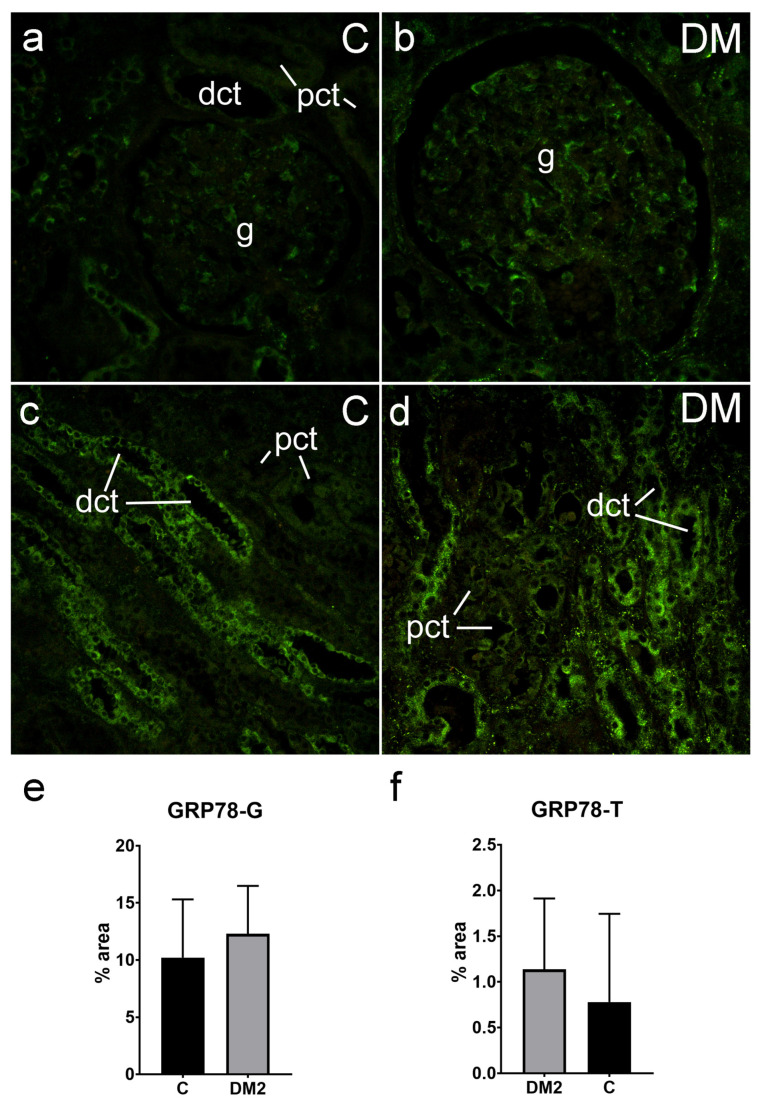
Immunohistochemistry expression of GRP78 (green) in renal tissues of nondiabetic and diabetic patients: C—control; DM2—diabetic patients; (**a**,**b**)—glomeruli (objective magnification ×40); (**c**,**d**)—tubule–interstitial compartment (objective magnification ×20); (**e**,**f**)—results of the GRP78 analysis—immunofluorescence signal was expressed as tissue section percentage area (% area)—in glomeruli (GRP78-G) and tubuli (GRP78-T) and compared using unpaired *T*-test. No significant difference was observed between compared groups. Grey column—diabetic patients (DM2); black column—control group of patients; mean (column) ± standard deviation (whisker) is presented. g—glomerulus; pct—proximal convoluted tubule; dct—distal convoluted tubule.

Extensive expression of GRP78 was found in the glomeruli, as well as in the tubule–interstitial compartment of the cortex of the studied patients. GRP78 expression was strong in the glomerular cells and in the epithelial cells of different tubules (Figure 7). Expression of GRP78 in glomeruli was 10.21 ± 510% for control and 12.29 ± 4.20% in diabetic patients. GRP78 expression in tubuli was 0.78 ± 0.97% for the control group and 1.14 ± 0.78% for the diabetic group. However, we failed to record any difference in the expression of GRP78 in the renal cortex, glomeruli (*p* = 0.330), or tubule–interstitial compartment (*p* = 0.111 for logarithmically transformed data) of the diabetic patients in comparison with the nondiabetic patients (Figure 7e,f).

## 3. Discussion

Despite intensive research on the role of autophagy in different biological processes, there are no data on the immunohistochemical expression of autophagy markers in the embryonic and fetal development of the human embryo. In this study, we aimed to investigate the expression of autophagy markers LC3B and LAMP2A and the ER stress marker GRP78 in diabetic kidney samples and compare them with samples from nondiabetic patients. In addition, in order to understand the potential role of autophagy and ER stress in the development of human kidney, we analyzed the expression of these markers in different renal structures during the embryonal and fetal periods.

LC3B is a structural component of the inner and outer layers of the autophagosome membrane [7,8], crucial for developing autophagosomes. Its expression has been used as an autophagy marker [6]. The moderate expression of LC3B that we found in the mesonephric glomeruli and tubules points to the role of macroautophagy processes in the formation and function of metanephric structures during early embryonal development. Accentuated LC3B immunoreactivity in the paramesonephric and mesonephric ducts, which was stronger in the 9/10th DW compared with the 7/8th DW, and the moderate LC3B expression that we also found in the mesenchyme surrounding the paramesonephric ducts in the 9/10th DW point to the role of macroautophagy in key processes of urogenital tract morphogenesis, during which cells of the mesonephric and paramesonephric ducts go through intensive processes of proliferation and remodeling. The expression pattern of LC3B in the fetal kidney (15/16th DW), with weak expression in the glomeruli and moderate expression in the developing tubules, in addition to the stronger expression in the renal medullary tubular structures, points to the more extensive role of macroautophagy in the remodeling of tubular epithelial cells, in comparison with podocyte and mesangial cell development. A similar pattern of expression was also found in the early postnatal kidney; however, postnatal LC3B expression was less intense, potentially due to slower remodeling processes in the almost-developed kidney, which demand less intense autophagy machinery. The presence of LC3B in the developing blood vessels during the fetal period was also expected, concerning intense vessel remodeling during renal tissue development and maturation. These results are in agreement with the results of a study conducted by Wen-jin et al. (2021), which used bioinformatics tools to find the expression of ATG genes in various populations of cells in the human fetal kidney, including podocytes, mesangial cells, nephron progenitor cells and different epithelial cells of a developing/developed nephron tubular system [27]. 

The transmembrane glycoprotein LAMP-2A plays the role of receptors on lysosomes [1,3]. This receptor plays an important role in CMA [11] by transferring cytosolic proteins to the lysosomes, which is particularly important in eliminating oxidized proteins [41,42]. It was found that the level of lysosomal LAMP2A is elevated upon CMA activation [26]. Substrate binding to LAMP2A is the rate-limiting step of the CMA processes. The level of LAMP2A at the lysosomal membrane, which is regulated by de novo synthesis, redistribution, and its degradation, is directly correlated with CMA activity [43,44,45,46]. A similar pattern of LAMP2A immunoexpression during renal development to LC3B points to the activation and role of CMA in the formation of mesonephric structures. In addition, the presence of LAMP2A in the mesonephric and paramesonephric ducts also points to the extensive CMA processes in cellular remodeling during urogenital system organogenesis. The moderate expression of LAMP2A that we observed in the glomeruli of the fetal kidney and the strong LAPM2A immunoreactivity in the developing tubuli, with a very strong presence in the medullary tubular structures, point to the intense CMA processes during the remodeling of tubular epithelial cells and nephron development and maturation. In the postnatal kidney, LAMP2A immunoreactivity was the strongest in the proximal convoluted tubules and the collecting duct epithelia, which might point to intense CMA processes in the functioning of these two structures. Namely, in PCT, the most intense re-absorption processes occur and lysosomal degradation of the re-absorbed proteins takes place [47,48]. Moreover, it was proven that CMA and LAMP2A are important in maintaining endocytotic machinery in proximal tubules by the regulation of megalin positioning in the apical membrane [49]. On the other hand, in the collecting ducts, constant dynamic remodeling of the aquaporin-made water pores occurs, which might also require the processes of autophagy degradation and protein recycling [50,51,52,53]. 

The endoplasmic chaperon protein GRP 78 is important in unfolded protein response during ER stress [16], which, in turn, is followed by autophagy activation. Lee and collaborators proved that GRP78 plays a role in the formation of autophagosomes and is necessary for stress-induced autophagy [17]. During embryonal development, the expression of GRP78 was strong in the mesonephric glomeruli and tubules, and moderate expression was also observed in the paramesonephric and mesonephric ducts, being more pronounced in the mesonephric than in the paramesonephric duct and the mesenchyme surrounding the mesonephric duct. These data point to the activation of the ER stress pathways during the development of mesonephric structures. Moreover, in the fetal kidney (during the 15/16th DW), strong GRP78 expression was found in all cortical and medullary structures, including the glomeruli, tubular structures, and developing blood vessels, pointing to an increase in the activation of the ER stress pathways with intense growth and development of the kidney. In the postnatal human kidney, the strongest expression of GRP78 was observed in the collecting duct epithelium, and moderate expression was found in other cortical and medullar structures. Again, this might be related to the intense activity of these cells with high energy expenditure and, consequently, a high level of cellular oxidative stress. 

As the most common complication of diabetes, DKD is strongly associated with chronic kidney disease (CKD) and is the leading cause of ESRD [54,55]. DKD’s pathophysiology is very complex, involving the activation of several different pathways. Since the activation of autophagy is crucial for the survival of kidney cells in critical conditions, such as hypoxia and oxidative stress [56], several studies have pointed to disturbances in the autophagy processes as some of the involved mechanisms [56,57,58,59,60]. Hence, we studied the expression of autophagy markers in the kidney tissues of diabetic patients and compared it with that in nondiabetic patients. We found moderate-to-weak LC3B expression, with occasional strong LC3B expression only in the glomeruli of nondiabetic patients. In addition, we found a significant decrease in the expression of LC3B in the glomeruli of the diabetic patients in comparison with the nondiabetic patients. Concerning the role of LC3B in autophagosome formation [6,7,8], it could be concluded that DM results in a decrease in macroautophagy processes in glomerular cells, which might involve podocytes, as well as mesangial cells. However, we did not find a difference in the expression of LC3B in the tubule–interstitial compartment of the cortex. These results are not in agreement with data on highly elevated LC3B-II/LC3B-I expression upon lipopolysaccharide (LPS) induction of acute kidney injury (AKI) in mice, which suggested the likely role of LC3B in repairing renal injury [61]. However, the development and progression of damage to the kidney by chronically increased blood sugar could not be directly compared with the mechanisms of the AKI. 

In addition, we studied the expression of LAMP2A in the kidney tissues of diabetic and nondiabetic patients. To the best of our knowledge, this is the first report on LAMP2A expression in kidneys from patients with DM2. LAMP2A immunoreactivity was mostly weak in the glomeruli, as well as in the tubule–interstitial compartment. However, we found strong LAMP2A immunoreactivity in the tubules of the diabetic patients. The expression of LAMP2A was significantly higher in the tubule–interstitial compartment of the diabetic patients in comparison with nondiabetic patients. However, there was no difference in the expression of LAMP2A in the glomeruli between the diabetic and nondiabetic patients. These data indicate the activation of intense CMA processes in the tubules of patients during chronic diabetes. Yuan et al. (2022) concluded that CKD is featured by a premature aging phenotype [43]. Aging-related CKD has been connected with a decrease in CMA, with a reduction in the abundance of LAMP2A, which leads to mitochondrial dysfunction [62,63]. This age-associated LAMP2A decrease is related to the instability and degradation of LAMP2A due to the lysosomal membrane instability [64], rather than the changes in LAMP2A itself [43,65,66]. In addition, a recent study has shown a connection between renal hypertrophy in rats with streptozotocin (STZ)-induced diabetes and reduced CMA with decreased levels of LAMP-2A in renal cortical cells [67]. The discrepancies between these results and the results of our study, in which we observed the activation of CMA in the renal tubules, could be attributed to the fact that in the mentioned study, acute effects of high hyperglycemia in the DM1 model were studied over 3–7 days, while in our study, we had specimens from long-term DM2 patients. In our study, patients with severe kidney failure were not included. The results of our study indicate that during chronic DM2 activation, CMA occurs in the renal cortical tubuli. This is in line with the increased activity of tubular cells in the higher degree of glucose reabsorption. However, damage to the tubular cells caused by different mechanisms involved in DKD pathogenesis [68,69] is expected to require an elevated level of degradation of damaged cellular components by CMA. 

The main limitation of our study is its small sample size and its retrospective and cross-sectional nature. Nevertheless, studies of human early embryonal fetal tissue are not common because of the exclusive nature of the material and its often poor preservation. Therefore, we believe that our results contribute to the knowledge of autophagy-related markers in human renal development. However, to determine the underlying mechanisms, prospective experimental studies are necessary, including knock-out mouse models with the specific deletion of particular factors in distinct cell populations, experimental interventions, or longitudinal monitoring. In addition, the next limitation is that we used only immunohistochemistry, not Western blot or mRNA analyses. Immunofluorescence signals are used widely in biological sciences to quantitate proteins (e.g., flow cytometry and image cytometry). Fluorescence microscopy also quantifies the spatial information of protein distribution in tissues or cells in addition to fluorescence. The latter is important for our results because we also aimed to reveal the protein distribution in different kidney structures, especially during the early embryonal/fetal development of the human kidney. Therefore, immunohistochemistry, which enables the detection of spatial distribution, was chosen as the most adequate method. Although these methods enable the quantification of proteins/gene products, with Western blot and qRT-PCR, spatial resolution is lost, which was of great importance in our research. In addition, we used an archive collection of sections of human embryos for the immunohistochemistry. The collection of material for Western blot and/or RT-PCR from human embryos in the early stage—especially structures in developing kidneys—is not possible due to related technical and ethical issues. Although these conclusions deserve further experimental confirmation, we believe that they provide good direction for future studies. 

GRP78 is an endoplasmic chaperon protein that plays a role in unfolded protein response during ER stress, involved in the return to ER homeostasis [16]. GRP78 plays a role in the formation of autophagosomes and is necessary for stress-induced autophagy, which is also activated in response to ER stress [17]. During the present study, we found the extensive expression of GRP78 in the glomeruli and the tubule–interstitial compartment of the cortex of studied patients, which was strong in the glomerular cells, as well as in the epithelial cells of different tubules. There was no difference in the expression of GRP78 between the renal cortex of the diabetic and nondiabetic patients. 

It is interesting to compare these data with a study in which authors determined the level of GRP78 in the serum of patients with DM2 [70]. Compared with the control group, the levels of circulating GRP78 were significantly higher in the DKD group. 

Liu et al. (2008) in a study on rats showed a significant increase in the expression of GRP78 in the kidney tissue of diabetic rats, as well as a slight immunoreactivity of this molecule in normal rats [71]. This finding suggests the activation of ER stress in diabetic rats. 

ER stress is triggered by various kidney diseases. In addition to diabetic nephropathy, these include renal fibrosis, inflammatory condition, osmolar contrast-induced renal injury, and ischemia–reperfusion [70].

However, the strong immunoexpression of GRP78 in the glomeruli and tubuli of both the diabetic and nondiabetic patients could be a reflection of the intense metabolic activities in renal cells, which are usually related to ER stress and the need for unfolded protein reparation. 

## 4. Materials and Methods

### 4.1. Tissue Procurement and Processing

Human conceptuses were obtained from the Department of Gynecology and Obstetrics and the Department of Pathology of the University Hospital of Split after spontaneous abortions or ectopic pregnancies. Approval of the Ethics and Medicines Committee of the Split University Hospital was obtained in accordance with the Declaration of Helsinki [72] (class: 003-08/16-03/0001, approval number: 2181-198-03-04-16-0024). The poorly preserved material was discarded. The age of the conceptuses was estimated based on the crown–rump length and Carnegie stages [73]. A total of 5 normal human conceptuses were collected between 7 and 16 weeks of development, while postnatal tissue was obtained from the autopsy of a healthy 1.5-year-old child. The tissue specimens were fixed in 4% paraformaldehyde in phosphate-buffered saline (PBS, pH 7.4).

In addition, we analyzed the renal tissue of 20 patients who had undergone nephrectomy due to renal carcinoma at the University Hospital of Split: 9 patients with diabetes, and 11 patients without diabetes. The Ethics Committee of the University Hospital of Split approved the study (class: 500-03/21-01/158, approval number: 2181-147/01/06/M.S.-21-02). The research included patients who, as of 1 January 2018. until 1 October 2021. were subjected to a nephrectomy due to renal cancer at the KBC Split Clinic, and the samples were analyzed and stored in the archives of the Department of Pathology, Forensic Medicine and Cytology, KBC Split. Clinical-diagnostic data were collected from the available documentation, and based on comorbidity data, the subjects were divided into two groups: patients with type 2 diabetes (N = 11) and patients without diabetes (N = 9). An immunohistochemical tissue analysis was performed on the archived kidney tissues for each subject. Inclusion criteria were the existence/nonexistence of diabetes mellitus type II (for DM/C groups, respectively). Exclusion criteria were other chronic diseases that affect renal function. Paraffin-embedded tissue blocks Normal tissue adjacent to the carcinoma was fixed in buffered 4% paraformaldehyde. Laboratory data at the time of nephrectomy were obtained from hospital records. Since DKD is rarely confirmed by needle biopsy, and its diagnosis is usually based on the presence of primary disease (DM) together with signs of renal disease, and renal biopsy is not indicated in healthy individuals (which means that it is difficult to obtain adequate control tissue), it is common research practice to obtain renal tissue samples from diabetic patients and adequate control tissue from nondiabetic patients in this particular way [74,75]. This type of sampling is usual in studies on diabetic kidney disease because there are very few situations in which renal biopsy of healthy people is performed and the material from autopsy is not appropriate due to intense autolysis. Even large public studies, for instance, TCGA, collected specimens and data about the sections of pathologically unchanged tissues obtained during tumor extraction surgery. Although this is not ideal, it is a standard that provides a good alternative for tissues from healthy people that would be unethical to collect surgically. Hence, this approach is usually applied in most studies of this type.

### 4.2. Immunohistochemistry Procedure

Tissue samples were dehydrated in ethanol, cleared in the xylene, and embedded in paraffin wax using a standard procedure. The paraffin blocks were cut into 5 μm thick sections, which were mounted on glass slides.

Subsequently, the sections were deparaffinized in xylene (3 × 10 min), rehydrated in decreasing concentrations of ethanol-in-water solutions (100% ethanol 2 × 10 min; 96% ethanol 1 × 5 min and 70% ethanol—1 × 5 min), and rinsed with distilled water. Heating in sodium citrate buffer (pH 6.0) for 30 min using a steam cooker was performed for the antigen retrieval. After cooling down to room temperature, the sections were washed in PBS (15 min). Protein block (ab64226, Abcam, Cambridge, UK) was applied to prevent unspecific antibody binding, and they were incubated for 20 min. After removal of the protein block, a combination of primary antibodies diluted in PBS (Table 2) was applied, followed by incubation with the primary antibodies in a humid chamber overnight. The antibodies used in this study were already validated in our laboratory and have been used for immunofluorescence on mice and human tissues in several previous studies [13,41,76,77]. The next morning, the sections were washed in PBS (3 × 5 min), and an adequate combination of secondary antibodies was applied and incubated for 1 h in a humid chamber (Table 2). After washing in PBS (3 × 5 min), the nuclei were stained with 4′6′-diamidino-2-phenylindole dihydrochloride (DAPI) (2 min exposure) and washed with distilled water, and the slides were cover-slipped (Immumount, Shandon, Pittsburgh, PA, USA). The exclusion of the primary antibody from the procedure resulted in no staining in the tissue.

### 4.3. Data Acquisition and Analysis

Sections were viewed using a BX51 microscope (Olympus, Tokyo, Japan) and captured using a cooled digital camera (DS-Ri2; Nikon, Tokyo, Japan) with NIS-Elements F software (version 5.22. 00). In order to quantify the immunoexpression of autophagy markers in the glomeruli of the kidneys from diabetic and nondiabetic patients, 15 visual fields were captured at an objective magnification of 40× and a constant exposure time. For analysis of the tubule–interstitial compartment, 10 nonoverlapping fields that contained cortical tubules at the 20× objective magnification were captured.

Photomicrographs were processed and analyzed using ImageJ software (version 1.54, National Institutes of Health, Bethesda, MD, USA). The red counter signal was subtracted from the images with green. Then, we applied a median filter with a radius of 15.0 pixels, and the threshold was adjusted using the “Triangle” thresholding algorithm. The percentage area under the fluorescence was determined using the Analyze Particles function. The entire visual field area was used for the analysis of the tubule–interstitial compartment. In order to analyze the glomeruli, particular structures were manually outlined and isolated using Adobe Photoshop (Adobe Inc., San Jose, CA, USA). Then, the photomicrographs were processed as described above, and the percentage area fraction was calculated. For the purpose of presentation, the background was deleted, and slight contrasting was carried out.

### 4.4. Statistical Analysis

We used PAST 3.22 software (Øyvind Hammer, Natural History Museum, University of Oslo, Oslo, Norway) for statistical analyses. The normality of the data distribution was assessed using the Shapiro–Wilk test. Non-normally distributed data were logarithmically transformed in order to obtain the normal distribution. The *t*-test for unequal variances was used to compare differences between the patient groups. A *p*-value of less than 0.05 was considered significant.

## 5. Conclusions

The presented data give us new information about the expression of LC3B, LAMP2A, and GRP78 during embryonic, fetal, and early postnatal development. The spatiotemporal expression of LC3B, LAMP2A, and GRP78 indicates the important role of autophagy in processes that are yet to be fully explained during the early stages of renal development.

Data on the expression of autophagy markers at different stages of human kidney development contribute to our better understanding of the role of autophagy in the complex processes of kidney development. These data have additional importance due to the otherwise rarely available and valuable human embryonic and fetal kidney samples.

In addition, according to our knowledge, this is the first report on the expression of LAMP2A in the kidneys of patients with DM2. LAMP2A expression is significantly higher in the tubule–interstitial compartment of diabetic kidneys than in nondiabetic ones. There was no difference in expression in the glomeruli. Our data suggest that disturbances in the autophagy processes in the glomeruli and tubuli of diabetic kidneys are important factors in the pathogenesis of diabetic kidney disease. With the necessary further research, autophagy may define a therapeutic site of action for future use in treating patients with DKD.

## Figures and Tables

**Table 1:** Patient age, sex, and serum creatinine data.

	N Male	N Female	Age—Years (Range)	Creatinine—μmol/L (Range)
All	14	6	65.9 ± 7.83 (44–78)	96.74 ± 35.48 (50–214)
Non- diabetic	8	3	66.27 ± 8.83 (44–75)	91.27 ± 22.12 (50–121)
Diabetic	6	3	65.44 ± 6.89 (55–78)	104.25 ± 49.25 (63–214)

**Table 2:** List of primary and secondary antibodies used.

	Antibody	Code No.	Host	Dilution	Source
Primary	Anti-LC3B	ab48394	Rabbit	1:100	Abcam, Cambridge, UK
	Anti-LAMP2A	ab18528	Rabbit	1:100	Abcam, Cambridge, UK
	Anti-GRP78	PA5-19503	Rabbit	1:300	Thermo Fisher Scientific, Waltham, MA, USA
	Anti-CD31/PECAM-1 (H3), Alexa fluor 546 conjugated	sc-376764	Mouse	1:50	Santa Cruz Biotechnology Inc., Santa Cruz, CA, USA
Secondary	Alexa Fluor^®^488 AffiniPure Anti- Rabbit lgG (H + L)	711-545-152	Donkey	1:400	Jackson Immuno Research Laboratories, Inc., Baltimore, PA, USA

## Data Availability

Data related to this study are contained within the Supplementary Materials.

## Supplementary Materials

The following supporting information can be downloaded at: https://www.mdpi.com/article/10.3390/ijms25179152/s1.
